# Examining Emotional Climates as a Function of Maternal Parenting Style: A Growth Model That Examines Authoritarian Beliefs and Emotional Expressivity During Parent–Child Interaction

**DOI:** 10.3390/ijerph23060727

**Published:** 2026-05-30

**Authors:** Heather J. Risser, Alexandra E. Morford

**Affiliations:** 1 Department of Psychiatry and Behavioral Sciences, Feinberg School of Medicine, Northwestern University, Chicago, IL 60611, USA; 2 Anschutz Medical Center, University of Colorado, Denver, CO 80045, USA; allie.morford@childrenscolorado.org

**Keywords:** parent attitudes, parent behavior, emotional expressivity, authoritarian, parent beliefs, parent–child interaction, parenting style, social–emotional learning

## Abstract

**Highlights:**

**Public health relevance—How does this work relate to a public health issue?**

- Many socio-emotional and emotion regulation difficulties develop due to an interaction between genetic and environmental factors.
- By better understanding environmental influences on early child development, we can identify modifiable targets to promote optimal socio-emotional function and wellbeing.

**Public health significance—Why is this work of significance to public health?**

- Parents are the largest contributors to their child’s development and an understated component of our public health infrastructure.
- By scaling parent support and knowledge about child development, we can optimize child socio-emotional development and wellbeing.

**Public health implications—What are the key implications or messages for practitioners, policy makers and/or researchers in public health?**

- Parents are eager to access tools that are customizable for their child.
- We can meet parents where they are by leveraging existing infrastructure through community-based programing to build parent capacity to optimize parent behavior to support evidence-based practices in promoting child development.

**Abstract:**

Parental emotional expressivity toward their child is an integral component of creating a family emotional climate, which is the primary context in which children develop social–emotional skills. The current study sought to empirically test Darling and Steinberg’s model that parent attitudes that make up parenting style effect parental emotional expressivity during parent–child interaction. Using longitudinal data from the NICHD Study of Early Child Care and Youth Development (SECCYD), the authors examined the compounding effects of maternal authoritarian attitudes measured soon after birth on maternal emotional expressivity toward their infant across three time points (child at 6, 15, and 24 months old). Hierarchical linear modeling analyses (HLMs) demonstrated that a mother’s (*n* = 1165, *M_age_* = 28.2 years) authoritarian attitudes were associated with both decreased positive expressivity and increased negative expressivity toward their child at 6 months of age. Mothers who held more authoritarian attitudes at baseline demonstrated an increased rate of growth in negative expressivity toward their child over time. Maternal race and income were also significantly associated with the linear rate of growth of negative expressivity over time but not in positive expressivity. This suggests that authoritarian attitudes measured when the child is 1 month old continue to impact parent behavior up to 23 months later. This pattern suggests a potential window for effective universal prevention efforts in promoting nurturing parent behavior and promoting positive parent–child relationships. A possible target of prevention intervention could be providing parents with components of a modularized emotion regulation curriculum. The content could help parents to regulate their negative expressivity toward the child and focus on the message they want to convey to the child related to the child’s specific behavior.

## 1. Introduction

Social–emotional skills are important to long-term success in all aspects of life, including social competence, emotional expressivity, and self-esteem. Parents are key contributors to their child’s social–emotional development [1,2]. Qualities of parental behavior during parent–child interactions are thought to influence children’s social–emotional functioning [3,4,5,6]. One salient quality of parental behavior during parent–child interactions is parental emotional expressivity [7]. Emotional expressivity (EE) refers to the behavioral manifestations of emotion such as facial expressions, utterances, articulations, and body language that communicate internal emotional and affective states [8].

Parents’ EE when interacting with their child contributes to the creation of the family emotional climate (i.e., the quality of the emotional environment within the parent–child context), which is often the primary context in which children learn to identify, manage, and express emotions [9]. Negative family emotional climates are risk factors for a series of maladaptive outcomes, including youth anxiety and depression [10]. As such, it is important to understand the predictors of parental EE toward the child, to ultimately promote parental behaviors that support a child’s optimal social emotional development and curtail parent behavior that may inhibit optimal development.

Additionally, there is great value in understanding the potentially compounding effect of early parental EE. As transactional and dynamic systems theories suggest (e.g., Patterson’s Coercion Theory), parent–child interactions are dynamic, transactional, reinforcing, and intensifying over time [11]. Thus, patterns in behaviors and functioning develop from recurring interactions, and the development of social–emotional functioning is no exception. Parents model social–emotional functioning through their EE toward the child, and the child in turn emulates the parents’ behavior. This reinforces the parent’s behaviors and attitudes toward the child, further contributing to the emotional climate.

Parent Emotional Expressivity and Parenting Style. One early predictor of emotional expressivity is likely the parenting style that an individual subscribes to. Darling and Steinberg posited that parenting styles are made up of a series of emotional expressions, and that these expressions convey parental beliefs and attitudes toward the child rather than attitudes about the child’s behavior [6]. As such, parenting styles are the expression of parents’ attitudes and beliefs. Therefore, it follows that early attitudes of parenting style are likely to influence parents’ emotional expressivity toward the child during parent–child interactions.

Baumrind’s three parenting styles (authoritative, authoritarian, and permissive) describe parents’ attitudes or beliefs about childrearing [12]. For the purposes of this study, we focused on the effects of authoritarian beliefs, as this parenting style has been shown to be associated with a myriad of adverse child outcomes, including deficits in cognitive and language development, increased risk of externalizing and internalizing symptoms, and decreased academic achievement [13,14,15,16,17,18,19]. Parents with authoritarian parenting attitudes are characterized by a lack of warmth and tend to engage in high levels of parental control. Authoritarian parents tend to be detached, discourage verbal interaction and child autonomy, value obedience, and use discipline as a means to control child behavior.

As Darling and Steinberg proposed, parent attitudes are expressed through negative and positive regard during parent–child interactions [6]. These attitudes create a lens through which parents judge their child’s behavior (attributions of child behavior), which ultimately influence how parents interact with their child [20,21,22]. Research conducted by Crouch and colleagues found that the pathway between authoritarian parenting attitudes and harsh parenting practices was explained by parents’ attributions of hostile intent (e.g., children misbehave because they are intentionally trying to be annoying) and parent negative emotionality [23]. For example, a parent with more authoritarian parenting attitudes may perceive a crying infant as a defiance of discipline rather than an expression of need and respond with more negative emotional expressivity (e.g., scowling or yelling).

Without intervention, attitudes that undergird parenting style are thought to be trait-like in their stability across time and consistency across child-rearing contexts [24,25]. Further, based on the transactional process of parent–child interactions, early parental attitudes should predict a compounding effect of parents’ emotional expressivity toward their child over time. This effect may intensify over time, as children develop more autonomy, a characteristic that may make authoritarian parents uncomfortable or reactive. It is key to investigate the effects of parental attitudes and emotional expressivity early on. To date, there has been no empirical study among new parents of whether early attitudes of parenting style influence parents’ emotional expressivity and how attitudes may predict emotional expressivity over time and contribute to the creation of an emotional context that informs child development [26].

As a salient aspect of emotional expressivity, it is also important to evaluate various influencing factors on parenting styles. Two factors that have been previously studied are (1) parent race and (2) parent income. In assessing these constructs, it is important to note that income and race may have a confounding relationship, and so it is important to evaluate these aspects both separately and together [27].

Race and Parenting Styles. Research has shown differences in parenting styles across different races. One study found that Black, Latinx, Asian, and Native American mothers tend to support more authoritarian parenting values than White mothers [28]. Interestingly, researchers found that the gap in authoritarian values between Black and White mothers increased as children aged from kindergarten to third grade [28]. Another study found that the relationship between Black parents and authoritarian values was still significant after controlling for socio-economic status [29].

Although studies have shown differences in parenting styles utilized across races, research findings are convergent that children of authoritarian parents, regardless of race, tend to have poorer outcomes than children of parents who tend to engage in flexible and moderate levels of parental control (authoritative parenting attitude). For example, McLoyd and Smith demonstrated that parental warmth moderated the relationship between coercive control and child behavior problems across African American, European American, and Latinx children [30]. Radziszewska, Richardson, Dent, and Flay found that across racial groups, adolescents with more authoritarian parents had more maladaptive outcomes (i.e., increased depressive symptoms and smoking behavior, decreased academic grades) relative to adolescents with less authoritarian parents [31]. While it is important to consider the social context and cultural meaning when evaluating the effects of parenting styles across racial groups, that is beyond the scope of this paper. However, as a first step, it will be important to understand whether differences exist early in childhood and how any differences may change over time to better understand the family emotional context in which children develop.

Income and Parenting Style. Direct studies of the relationship between parenting styles and income are limited. There is some evidence of a common association across various populations between lower income and more authoritarian attitudes [32,33,34]. That said, the negative effects of authoritarian parenting styles seem to transcend socio-economic status, and there is consistent support across various populations that authoritarian parenting attitudes and behaviors are risk factors for maladaptive child development [35,36,37].

Child Mood. Given the transactional nature of parent–child interactions, it is unsurprising that child mood has been linked to parents’ behavioral responses. Children’s negative mood has been associated with parent outcomes such as decreased sensitivity, increased intrusive control, and harsh and punitive parenting [38,39,40]. To better isolate the effects of parent attitudes on emotional expressivity, it is important to account for, or control for, the possible influence of child negative mood.

The Current Study. The current study sought to empirically test Darling and Steinberg’s model that parent attitudes of parenting style effect parental emotional expressivity during the parent–child interaction [6]. To our knowledge, no study to date has longitudinally tested this model among parents with infants. Therefore, we examined the effects of maternal authoritarian attitudes on maternal emotional expressivity (positive and negative) toward their infant and how these relationships (or patterns) would change (or intensify) over time. Using longitudinal data and hierarchical linear modeling analyses (HLMs), we examined these relationships across three time points (i.e., child at 6, 15, and 24 months old). The rationale for this model was to assess whether any change in parental expressivity could be attributed to parental beliefs measured right after the child was born.

Hypotheses. Consistent with Darling and Steinberg’s model, we expected that maternal attitudes about authoritarian beliefs would be associated with maternal emotional expressivity toward their child [6]. Specifically, we predicted that higher authoritarian attitudes would be associated with lower levels of positive expressivity and higher levels of negative expressivity at baseline assessment. Furthermore, we expected that authoritarian parenting beliefs would predict growth trajectories of positive and negative expressivity over time, such that higher levels of authoritarian attitudes would be associated with decreasing positive and increasing negative expressivity toward the child over time. Finally, given that the effects of parenting style transcend ethnicity and income as previously mentioned, we expected that the relationship between authoritarian attitudes would not be different across racial groups or income levels [30,41].

## 2. Materials and Methods

The National Institute of Child Health and Human Development (NICHD) conducted the Study of Early Child Care and Youth Development (SECCYD). A total of 1364 mothers and their infants were recruited from hospitals in 10 major cities in the United States to participate in a longitudinal study (University of Arkansas at Little Rock; University of California at Irvine; University of Kansas; University of Pittsburgh; Temple University; University of Virginia; University of Washington at Seattle; Western Carolina Center and University of North Carolina at Chapel Hill; Wellesley College; and University of Wisconsin at Madison). A comprehensive description of the recruitment procedures, enrolled sample, study design, and measures can be found in the NICHD Early Child Care Research Network [42]. The analyses for this study used data obtained when the child was 6, 15, and 24 months old. Of the families enrolled in the SECCYD study, 1165 families with complete data at all three time points were used in the current analyses (85% of the initial enrolled sample).

The sample used in the current study consisted of White (86.6%) and Black (13.4%) mothers (*M* age = 28.2, *SD* = 5.7). The majority of the children in the sample were White (80.8%) or Black (12.5%) with other (6.7%). About half of the children that participated in the study were first born (44.7%), 34.8% were the second child, and 20.4% were the third through the seventh child. The child’s birth order differed by race, *Χ*^2^ (6) = 44.57, *p* < 0.001. Forty-five percent of children of White mothers were first born, with 41% for Black mothers, 37% of children of White mothers were the second child, with 22% for Black mothers, 13% of children of White mothers were the third child, with 25% of Black mothers, and 5% of children of White mothers were the fourth through the seventh child, with 12% for Black mothers. The mean total family income was $37,947.77, and the median total family income was $30,000.

### 2.1. Procedure

Mothers completed questionnaires, participated in interviews, and were observed interacting with their infants at several points during their child’s development. A subset of the variables measured in the SECCYD were used in the present study to examine the relationship between maternal attitudes about parenting style and maternal emotional expressivity toward the child during parent–child interactions. Maternal authoritarian attitudes were measured through a questionnaire when the child was 1 month old. When the child was 6, 15, and 24 months old, mother–infant interactions were video-taped over a 44 min cycle in either the family’s home or in a child-care setting (if used). At each time point, video-taped mother–infant interactions were observed and rated for maternal positive and negative expressivity toward the child. For a complete description of the observational techniques created for this study (Observational Ratings of the Caregiving Environment; ORCE), please refer to the NICHD Early Child Care Research Network [42,43,44].

### 2.2. Measures

Maternal attitudes about parenting styles were analyzed. The Traditional subscale of the Parental Modernity Scale of Child-Rearing and Educational Beliefs was administered to mothers when their child was 1 month old [45]. The self-report measure is a 22-item (out of a total of 30 items) questionnaire and was designed to assess the degree to which a parent endorses a traditional, inflexible, authoritarian view of parenting. The instrument reflects attitudes consistent with authoritarian beliefs, such as child behavior should follow adult directives, children’s autonomy should be limited, and children should not express their own ideas. Parents were asked to indicate the degree to which they agreed with the items using a 5-point Likert scale ranging from “Strongly Agree” to “Strongly Disagree”. The internal consistency for the sample used in these analyses was Cronbach’s *α* = 0.90 (the same value as the entire NICHD sample).

Maternal expressivity (positive and negative regard) toward their child was analyzed. Maternal expressivity for the child was rated from video-taped, semi-structured parent–child interactions by independent observers. Maternal positive regard for the child represents the observers rating of the mother’s expressions of positive feelings toward the child during the interaction. The mother’s demonstrations of warmth, acceptance, enthusiasm, physical affection, positive facial expressions, laughing with the child, praising the child and play are representative of positive regard for the child. Maternal negative regard for the child represents the observer’s rating of the mother’s expressions of negative feelings toward the child during the interaction. The mother’s demonstrations of disapproval, harshness, sarcasm, negative vocal tone, tense facial posture and strained expression, threatening, and excessive roughness are representative of negative expressivity toward the child. The observer’s ratings of maternal emotional expressivity (two separate scores for positive and negative regard) ranged from 1 (not at all characteristic of the mother) to 4 (highly characteristic of the mother). Correlations between positive and negative regard for the same observation ranged from *r* = −0.226 to *r* = −0.366 in this sample.

Child negative mood was analyzed. Child negative mood was rated from the same ORCE sessions described above. Child negative mood represents observer ratings of the extent to which the child expresses discontent by crying, fussing, frowning, tensing his/her body, or other expressions of negative mood. The observer’s ratings of child’s negative mood ranged from 1 (not at all characteristic of the child) to 4 (highly characteristic of the child). These ratings were made when the child was 6, 15, and 24 months old. For the purposes of this study, child negative mood was used only as a control in the analysis of maternal expressivity.

### 2.3. Statistical Analyses

To analyze the effects of a mother’s authoritarian attitudes on maternal expressivity toward their child over time, hierarchical linear modeling (HLM) was conducted using HLM version 6.06 [46]. HLM is ideal for evaluating the SECCYD data, which includes repeated variables, collected at uneven intervals of time, and individually varying number of data points [47].

First, the analyses modeled growth in both maternal positive and negative regard for the child as a linear function of time. Second, the analyses modeled the effect of parent attitudes on growth in both maternal positive and negative regard for the child. Third, the analyses modeled the effect of maternal race and income on growth in both maternal positive and negative regard for the child. Finally, the second and third analyses were conducted again but controlled for child negative mood.

## 3. Results

Mothers demonstrated differences in both positive and negative expressivity for the child with observer ratings ranging from 1 to 4. Means and standard deviations for each time period for both positive and negative regard are reported in Table 1.

### 3.1. Maternal Positive Expressivity

The basic random effects model (Model 1a) demonstrated individual variation among ratings of mothers’ positive regard toward their children during mother–child interactions when their child was 6 months old, *VAR (r*_0*i*_*)* = 0.13, *p* < 0.001. In the unconditional linear growth model (Model 1b) time was added as a predictor, centered at 6 months. Results demonstrated significant individual variation among ratings of a mother’s positive regard for their child during mother–child interactions when the child was 6 months old, *VAR (r*_0*i*_*)* = 0.14, *p* < 0.001, and in linear growth, *VAR (r*_1*i*_*)* = 0.03, *p* < 0.001 (see Table 2).

The reliability for the individual-level intercept was π_0i_, *r_xx_*^′^ = 0.36, and the reliability for the linear slope was π1i, *r_xx_*^′^ = 0.15, indicating that the individual’s intercept and slope were important to take into account in the analysis, i.e., keeping the coefficients randomly varying. Although analysis of the fixed effects did not demonstrate significant linear growth over time, *β*_10_ = −0.01, *p* = 0.38, the correlation between a mother’s positive regard toward their child when the child was 6 months and rate of linear growth, *r* = −0.26, *p* < 0.001, was significant. The negative correlation suggests that mothers who received higher ratings of positive regard toward their children in the 6-month interaction demonstrated weaker linear growth from 6 to 24 months than mothers who received lower ratings of positive regard toward their children in the 6-month interaction. When the time-varying covariate negative child mood (grand mean centered; Model 1c) was added to the model, results still demonstrated individual variation among ratings of mothers’ positive regard for their children during mother–child interactions when their child was 6 months old, VAR (r_0i_) = 0.14, *p* < 0.001, and in linear growth, VAR (r_1i_) = 0.03, *p* < 0.001 (see Table 2). Analysis of the fixed effects demonstrated a significant effect of the child’s negative mood with the mother’s positive regard over time, *β*_20_ = −0.09, *p* < 0.001, but there was still not a significant linear change in positive regard over time when controlling for child’s negative mood, *β*_10_ = −0.01, *p* = 0.51.

Conditional random effects growth models were also examined. Model 1c demonstrated that the child’s negative mood was related to maternal behavior during interactions. Thus, the effects of parenting beliefs on maternal positive regard were examined while controlling for child negative mood. The first of these models included maternal authoritarian parenting beliefs when the child was 1 month old (Model 1d) to examine whether authoritarian beliefs would provide a more accurate estimation of maternal positive regard for the child. The second model included maternal authoritarian beliefs when the child was 1 month old and maternal race (Model 1e) to examine whether race accounted for additional variance above authoritarian beliefs in predicting maternal positive regard for the child. Because race was associated in a preliminary ANOVA, the third model included maternal authoritarian beliefs and maternal race (Model 1f).

Authoritarian attitudes were analyzed. The traditional authoritarian subscale was used for the analysis as randomly varying and grand mean centered. Authoritarian attitudes were significantly related to ratings of maternal positive regard at 6 months after controlling for child negative mood, *β*_01_ = −0.01, *p* < 0.001. Thus, positive regard decreases by 0.01 with each 1-point increase on the authoritarian belief subscale when the child’s negative mood is average. Furthermore, 28.2% of the variance, τ_00_, in average positive regard at 6 months was accounted for by authoritarian beliefs. Authoritarian beliefs also accounted for 3.7% of the variance, τ_11_, in the linear growth in maternal positive regard. The average growth rate in ratings of maternal positive regard from 6 to 24 months, while controlling child negative mood, was not significant, *β*_10_ = −0.01, *p* = 0.47. The interaction between authoritarian beliefs and rate of growth was not significant, *β*_11_ = 0.00, *p* = 0.45. The interaction between authoritarian beliefs of the mother and the negative mood of the child on positive regard was not significant, *β*_21_ = −0.00, *p* = 0.07.

Maternal race was analyzed. Race did not predict maternal positive regard at 6 months, *β*_02_ = 0.08, *p* = 0.16, suggesting that mothers who identified as White, relative to mothers who identified as Black, received equivalent ratings of positive regard for their children during interactions with their child at 6 months of age. The linear growth rate in positive regard when authoritarian beliefs were average did not differ for mothers who identified as White relative to those who identified as Black, *β*_12_ = 0.08, *p* = 0.08. Together, authoritarian beliefs and race accounted for 28.1% of the variance in average positive regard at 6 months and 3.9% in slope.

Authoritarian beliefs by maternal race were analyzed. The authoritarian beliefs by maternal race interaction for maternal positive regard at 6 months was not significant, *β*_03_ = 0.00, *p* = 0.98. The authoritarian beliefs by maternal race interaction for linear growth in maternal positive regard was not significant, *β*_13_ = 0.00, *p* = 0.53.

Income was also analyzed. Income predicted positive regard at 6 months, *β*_04_ = 0.00, *p* < 0.01, suggesting that mothers who report higher income relative to those who report lower income are rated higher in positive regard during interactions with their 6-month-old infants. The linear growth rate in positive regard when authoritarian beliefs were average did not differ by income, *β*_14_ = 0.00, *p* = 0.65.

Income by race was analyzed. There were no significant income by race interactions at 6 months, *β*_05_ = −0.00, *p* < 0.47, or for linear growth, *β*_15_ = −0.00, *p* = 0.75.

### 3.2. Maternal Negative Regard

The basic random effects model (Model 2a) demonstrated individual variation among ratings of the mother’s negative regard toward their child during mother–child interactions when the child was 6 months old, *VAR (r*_0*i*_*)* = 0.03, *p* < 0.001. In the unconditional linear growth model (Model 2b) time was added as a predictor, centered at 6 months. Results demonstrated significant individual variation among ratings of mothers’ negative regard for their children during mother–child interactions when their child was 6 months old, *VAR (r*_0*i*_*)* = 0.01, *p* < 0.001, and in linear growth, *VAR (r*_1*i*_*)* = 0.04, *p* < 0.001 (see Table 2). Concomitantly, the reliability for the individual-level intercept, π_0i_, was *r_xx_*^′^ = 0.15, and the reliability for the linear slope, π_1i_, was *r_xx_*^′^ = 0.47, indicating that the individual’s intercept and slope were important to take into account in the analysis, i.e., keeping the coefficients randomly varying. Analysis of the fixed effects demonstrated significant linear growth over time, *β*_10_ = 0.08, *p* < 0.001, and there was a negative correlation between mothers’ negative regard for their children when the child was 6 months and the rate of linear growth, *r* = −0.35, *p* < 0.001. The negative correlation suggests that mothers who received higher ratings on negative regard for their child at 6 months demonstrated less linear growth of negative regard toward their child from 6 to 24 months than mothers who received lower ratings on negative regard for their child at 6 months. When the time-varying covariate, negative child mood (Model 2c), was added to the model, results demonstrated individual variation among ratings of mothers’ negative regard for their child during mother–child interactions when the child was 6 months old, *VAR (r*_0*i*_*)* = 0.01, *p* < 0.001, and in linear growth, *VAR (r*_1*i*_*)* = 0.04, *p* < 0.001 (see Table 3). Analysis of the fixed effects demonstrated a significant effect of the child’s negative mood with the mother’s negative regard over time, *β*_20_ = 0.12, *p* <.001, and significant linear growth over time in the mother’s negative regard controlling for the child’s negative mood, *β*_10_ = 0.07, *p* < 0.001.

Conditional random effects growth models were also examined. Model 2c demonstrated that the child’s negative mood impacts maternal behavior during interactions. Thus, the effects of parenting beliefs on maternal negative regard were examined while controlling for child negative mood. The first of these models included maternal authoritarian parenting beliefs when the child was 1 month old (Model 2d) to examine whether authoritarian beliefs would provide a more accurate estimation of maternal negative regard toward the child. The second model included maternal authoritarian beliefs when the child was 1 month old and race (Model 2e) to examine whether race accounted for additional variance above authoritarian beliefs in predicting negative regard for the child. Because race was associated in a preliminary ANOVA, the third model included maternal authoritarian beliefs and maternal race (Model 2f).

Authoritarian beliefs were analyzed. Authoritarian beliefs were significantly related to ratings of maternal negative regard at 6 months after controlling for child negative mood, *β*_01_ = 0.002, *p* = 0.001. Thus, negative regard increases by 0.002 with each 1-point increase on the authoritarian beliefs subscale when the child’s negative mood is average. Furthermore, 9.0% of the variance, τ_00_, in average negative regard at 6 months was accounted for by authoritarian beliefs. Authoritarian beliefs also accounted for 6.7% of the variance in the linear growth in maternal negative regard, τ_11_. The average growth rate in ratings of maternal negative regard from 6 to 24 months while controlling for child negative mood was positive, *β*_10_ = 0.08, *p* < 0.001 (i.e., a 9-month increase in child age was associated with a 0.08 increase in negative regard). The interaction between authoritarian beliefs and rate of growth was significant, *β*_11_ = 0.003, *p* < 0.001, indicating that the growth in negative regard over 9 months increased by 0.003 for mothers with each 1-point increase in score on the authoritarian beliefs subscale. Additionally, there was a significant effect of traditional authoritarian beliefs on the child negative mood–maternal negative regard relationship, *β*_21_ = 0.003, *p* < 0.01, indicating that a 1-point increase on the authoritarian beliefs subscale was associated with a 0.003 increase in the relationship between the baby’s negative mood and the maternal negative regard relationship.

Maternal race was analyzed. Although race did not predict maternal negative regard at 6 months, *β*_02_ = −0.01, *p* = 0.88, the linear growth rate in negative regard when authoritarian beliefs were average was steeper for mothers who identified as Black relative to those who identified as White, *β*_12_ = −0.17, *p* < 0.001. The interaction between authoritarian beliefs and rate of growth remained significant with race in the model, *β*_11_ = 0.002, *p* < 0.05. Together, authoritarian beliefs and race accounted for 10.4% of the variance in average negative regard at 6 months and 13.3% in slope.

Authoritarian beliefs by maternal race were analyzed. The authoritarian beliefs by maternal race interaction for maternal negative regard at 6 months was not significant, *β*_03_ = −0.00, *p* = 0.78. The authoritarian beliefs by maternal race interaction for linear growth in maternal negative regard was not significant, *β*_13_ = −0.00, *p* = 0.51.

Income was analyzed. Income predicted negative regard at 6 months, *β*_04_ = −0.00, *p* = 0.001, suggesting that mothers who report higher income relative to those who report lower income are rated lower in negative regard during interactions with their 6-month-old infants. The linear growth rate in negative regard when authoritarian beliefs were average did not differ by income level, *β*_14_ = 0.00, *p* = 0.67.

Income by race was analyzed. There were no significant income by race interactions at 6 months, *β*_05_ = 0.00, *p* = 0.22, or for linear growth, *β*_15_ = 0.00, *p* = 0.29.

## 4. Discussion

Parental emotional expressivity toward their child is an integral component of creating a family emotional climate, which is the primary context in which child development of social–emotional skills occur. As deficits in social–emotional functioning have far-reaching maladaptive outcomes, and the investigation of risk factors to parent emotional expressivity is paramount to informing parenting programs. One salient and malleable predictor of family emotional climate is parental emotional expressivity. Informed by transactional and dynamic system theories such as Coercion Theory, we examined the potential compounding effect of maternal attitudes on maternal expressivity across three time points and the potential influence of maternal race and income on this relationship [11].

The present results support the proposition that parenting style conveys parental attitudes toward their child. Maternal attitudes about parenting style measured soon after the child was born predicted maternal expressivity toward the child over the subsequent 24 months. Consistent with previous research that has shown that authoritarian parenting attitudes are associated with parent negative emotionality, we found support for the hypothesis that maternal authoritarian attitudes would be associated with maternal emotional expressivity when the baby was 6 months old [23]. As predicted, we found that authoritarian attitudes were associated with both decreased positive expressivity and increased negative expressivity toward the child at 6 months old. We also found that, consistent with our hypotheses, authoritarian attitudes moderated the linear rate of growth in negative expressivity toward the child, such that mothers who held more authoritarian attitudes when their child was 1 month old demonstrated an increased rate of growth in negative expressivity toward their child over time. Interestingly, authoritarian attitudes did not moderate the linear rate of growth in positive expressivity.

This pattern of results suggests that authoritarian beliefs not only contribute to more negative expressivity when the child is 6 months old but also contribute to increasing negativity expressed during parent–child interactions that is not accompanied by a parallel increase in positivity. Thus, authoritarian attitudes appear to distinguish parenting behaviors that may be interpreted as negative feelings toward the child in particular and not just increasingly salient feelings regardless of valence.

Based on extant research, we expected that the results would not differ across racial groups or income levels [28,30,31]. Although positive and negative expressivity toward the child did not differ significantly between maternal races when children were 6 months old, the linear growth rate in negative expressivity was steeper for Black mothers relative to White mothers. These results suggest that maternal race is significantly associated with the linear rate of growth of negative expressivity over time but that there is not a parallel interaction in positive expressivity over time. Thus, maternal race does not appear to be a distinguishing factor of maternal negative expressivity toward the child when the child is 6 months old but may be influential as the child ages. Although this study provides preliminary support for the idea that race may influence the relationship between authoritarian attitudes and negative expressivity, further research is needed to explore the mechanism of influence and how the racial difference in rate of negative expressivity impacts child behavior and long-term outcomes. For example, observer ratings of positive and negative regard may have included racial bias, or contextual factors such as racism could impact parent behavior in an effort to keep children safe in communities impacted by historic and systemic racism.

The effect of income on positive and negative expressivity speaks to the potential protective effect of high income. The current results indicate that higher income was related to both higher positive and lower negative maternal expressivity when the child was 6 months old. Given the often confounded relationship between race and income, future studies should further explore the independent and shared influence of income and race on maternal expressivity toward the child.

### Strengths, Limitations and Future Directions

Although parenting style has been described as trait-like in its stability, given the transactional nature of parenting, child characteristics and aspects of parent behavior may contribute to altering authoritarian beliefs and parental emotional expressivity. That is, parent–child interactions and parent’s emotional reactions to their child’s emotions and behavior may contribute to the development or revision of both parenting beliefs and emotional expressivity. Therefore, only measuring authoritarian beliefs at one time point poses a limitation to capturing the potential dynamic and transactional nature of parenting beliefs and the impact of beliefs on positive and negative regard. Additionally, the dynamic nature of the parent–child relationship and other impacts on parent development can impact the evolution of both parental beliefs and parental emotional expressivity independent of the variables measured in this study. Future research could assess parenting beliefs at multiple time points concurrent with rating positive and negative regard within a context of multiple potential covariates such as parent age, fertility, additional children, and other aspects of parent–child relationships.

Another potential limitation of these findings is the lack of wider contextual factors such as the impact of social policies on individual factors like race and income. Additionally, the potential confounding of race and income could account for variance due to intersectionality. Mothers who identified as Black relative to mothers who identified as White reported significantly lower income. Future research could examine the impact of authoritarian beliefs on maternal behavior in families matched by income. It would also be important to assess racial identity within the context of racism, discrimination, and safety, as these contextual impacts on race could influence parenting behavior. Future research could also assess older children to examine the effect of authoritarian beliefs and positive and negative regard in latency-aged or adolescent children.

## 5. Conclusions

This study demonstrates that parents who endorse attitudes consistent with an authoritarian parenting style also demonstrate less positive and more negative expressivity for their child during parent–child interactions when the child is 6 months old. This suggests that authoritarian attitudes measured when the child is 1 month old may continue to impact parent behavior up to 5 months later. Furthermore, authoritarian attitudes contribute to growth in negative expressivity toward the child at 15 and 24 months, suggesting that attitudes measured when the child is 1 month old continue to impact parent behavior up to 23 months later. This pattern may suggest a potential window for effective universal prevention efforts in promoting nurturing parent behavior and promoting positive parent–child relationships. A possible target of prevention intervention could be providing parents with components of a modularized emotion regulation curriculum. The content could help parents to regulate their negative expressivity toward the child and focus on the message they want to convey to the child related to the child’s specific behavior. Parents serve as one of the largest and most significant aspects of our public health infrastructure for children and youth. Universal prevention strategies aimed at building parent capacity to promote optimal child development has the potential to provide a scalable approach to supporting youth social–emotional wellbeing.

## Figures and Tables

**Table 1:** Means and Standard Deviations for Maternal Positive and Negative Expressivity at 6-, 15- and 24-months.

Time Period	Positive Expressivity (Regard)		Negative Expressivity (Regard)	
	*M*	*SD*	*M*	*SD*
6-months	2.85	0.67	1.09	0.33
15-months	2.81	0.66	1.06	0.29
24-months	2.83	0.71	1.25	0.57

**Table 2:** Fixed and random effects for growth models for positive expressivity (regard).

Fixed Effects	Model 1a	Model 1b	Model 1c	Model 1d	Model 1e	Model 1f	Model 1g	Model 1h
Intercept	2.84 ***	2.85 ***	2.85 ***	2.85 ***	2.77 ***	2.77 ***	2.80 ***	2.83 ***
Linear Growth		−0.01	−0.01	−0.01	−0.08	−0.06	−0.05	−0.05
Child Negative Mood			−0.09 ***	−0.07 ***	−0.10 *	−0.12	−0.11	−0.04
Authoritarian Beliefs				−0.01 ***	−0.01 ***	−0.01 **	−0.01 **	−0.01 **
Authoritarian Beliefs x Time				0.00	0.00	−0.00	−0.00	−0.00
Authoritarian Beliefs x Child Mood				−0.00	−0.00	−0.00	−0.00	−0.00
Maternal Race					0.08	0.08	0.05	0.02
Maternal Race x Time					0.08	0.06	0.06	0.05
Maternal Race x Child Mood					0.05	0.05	0.04	−0.03
Authoritarian Belief x Maternal Race						0.00	0.00	0.00
Authoritarian Belief x Maternal Race x Time						0.00	0.00	0.00
Authoritarian Belief x Maternal Race x Child Mood						−0.00	−0.00	−0.00
Income							0.00 **	0.00
Income x Time							0.00	0.00
Income x Child Mood							0.00	0.00
Income x Maternal Race								−0.00
Income x Maternal Race x Time								−0.00
Income x Maternal Race x Child Mood								−0.00
**Random Effects**	**Model 1a**	**Model 1b**	**Model 1c**	**Model 1d**	**Model 1e**	**Model 1f**	**Model 1g**	**Model 1h**
Intercept, *VAR (r*_0_*)*	0.13 ***	0.14 ***	0.14 ***	0.10 ***	0.10 ***	0.10 ***	0.10 ***	0.10 ***
Linear Growth, *VAR (r*_1_*)*		0.03 ***	0.03 ***	0.03 ***	0.03 ***	0.03 ***	0.03 ***	0.03 ***
Level-1 error, *VAR (e)*	0.33	0.30	0.30	0.30	0.30	0.30	0.30	0.30
	*n* = 1165	*n* = 1165	*n* = 1165	*n* = 1165	*n* = 1165	*n* = 1165	*n* = 1165	*n* = 1165

Note. All models are age-centered at 6 months. *** *p* < 0.001, ** *p* < 0.01, * *p* < 0.05.

**Table 3:** Fixed and random effects for growth models for negative expressivity (regard).

Fixed Effects	Model 2a	Model 2b	Model 2c	Model 2d	Model 2e	Model 2f	Model 2g	Model 2h
Intercept	1.12 ***	1.05 ***	1.05 ***	1.05 ***	1.05 ***	1.06 ***	1.04 ***	1.02 ***
Linear Growth		0.08 ***	0.08 ***	0.08 ***	0.23 ***	0.20 ***	0.20 ***	0.18 ***
Child Negative Mood			0.12 ***	0.10 ***	0.10 *	0.20 **	0.19 **	0.13 *
Authoritarian Beliefs				0.00 **	0.00 **	0.00	0.00	0.00
Authoritarian Beliefs x Time				0.00 ***	0.00 *	0.00	0.00	0.00
Authoritarian Beliefs x Child Mood				0.00 **	0.00 **	−0.00	−0.00	−0.00
Maternal Race					−0.01	−0.01	0.00	0.02
Maternal Race x Time					−0.17 ***	−0.15 **	−0.15 **	−0.13 **
Maternal Race x Child Mood					0.00	−0.09	−0.08	−0.02
Authoritarian Belief x Maternal Race						−0.00	−0.00	−0.00
Authoritarian Belief x Maternal Race x Time						−0.00	−0.00	−0.00
Authoritarian Belief x Maternal Race x Child Mood						0.01 **	0.01 **	0.01 **
Income							−0.00 **	−0.00
Income x Time							0.00	−0.00
Income x Child Mood							−0.00	−0.00
Income x Maternal Race								0.00
Income x Maternal Race x Time								0.00
Income x Maternal Race x Child Mood								0.00
**Random Effects**	**Model 2a**	**Model 2b**	**Model 2c**	**Model 2d**	**Model 2e**	**Model 2f**	**Model 2g**	**Model 2h**
Intercept, *VAR (r*_0_*)*	0.03 ***	0.01 ***	0.01 ***	0.01 **	0.01 **	0.01 **	0.01 **	0.01 **
Linear Growth, *VAR (r*_1_*)*		0.04 ***	0.04 ***	0.04 ***	0.03 ***	0.03 ***	0.03 ***	0.03 ***
Level-1 error, *VAR (e)*	0.14	0.09	0.09	0.09	0.09	0.09	0.09	0.09
	*n* = 1165	*n* = 1165	*n* = 1156	*n* = 1156	*n* = 1156	*n* = 1156	*n* = 1165	*n* = 1165

Note. All models are age-centered at 6 months. *** *p* < 0.001, ** *p* < 0.01, * *p* < 0.05.

## Data Availability

Data from the original study are publicly available with permission from the Inter-university Consortium for Political and Social Research (ICPSR). Data can be accessed by navigating to: https://www.icpsr.umich.edu/web/ICPSR/series/00233. (The data used in this study was accessed via in-person training June 2004, Chapel Hill, NC, USA). No new data was created for this project. Restrictions apply to the availability of these data.
